# N-acetylglucosamine 6-Phosphate Deacetylase (nagA) Is Required for N-acetyl Glucosamine Assimilation in *Gluconacetobacter xylinus*

**DOI:** 10.1371/journal.pone.0018099

**Published:** 2011-06-02

**Authors:** Vikas Yadav, Bruce Panilaitis, Hai Shi, Keiji Numuta, Kyongbum Lee, David L. Kaplan

**Affiliations:** 1 Department of Biomedical Engineering , Tufts University, Medford, Massachusetts, United States of America; 2 Department of Chemical and Biological Engineering, Tufts University, Medford, Massachusetts, United States of America; University of Minho, Portugal

## Abstract

Metabolic pathways for amino sugars (N-acetylglucosamine; GlcNAc and glucosamine; Gln) are essential and remain largely conserved in all three kingdoms of life, i.e., microbes, plants and animals. Upon uptake, in the cytoplasm these amino sugars undergo phosphorylation by phosphokinases and subsequently deacetylation by the enzyme *N*-acetylglucosamine 6-phosphate deacetylase (nagA) to yield glucosamine-6-phosphate and acetate, the first committed step for both GlcNAc assimilation and amino-sugar-nucleotides biosynthesis. Here we report the cloning of a DNA fragment encoding a partial nagA gene and its implications with regard to amino sugar metabolism in the cellulose producing bacterium *Glucoacetobacter xylinus* (formally known as *Acetobacter xylinum*). For this purpose, nagA was disrupted by inserting tetracycline resistant gene (nagA::tet^r^; named as ΔnagA) via homologous recombination. When compared to glucose fed conditions, the UDP-GlcNAc synthesis and bacterial growth (due to lack of GlcNAc utilization) was completely inhibited in nagA mutants. Interestingly, that inhibition occured without compromising cellulose production efficiency and its molecular composition under GlcNAc fed conditions. We conclude that nagA plays an essential role for GlcNAc assimilation by *G. xylinus* thus is required for the growth and survival for the bacterium in presence of GlcNAc as carbon source. Additionally, *G. xylinus* appears to possess the same molecular machinery for UDP-GlcNAc biosynthesis from GlcNAc precursors as other related bacterial species.

## Introduction

*N*-acetylglucosamine (GlcNAc) is a major component of structural polymers in bacteria, plants, and animals [1]. Chitin, a homopolymer of GlcNAc, is a structural material in many invertebrates, bacteria, fungi and algae (especially some diatoms) [2]. However, both gram-positive and gram-negative bacteria contain GlcNAc as a main constituent of their cell wall peptidoglycan. Since GlcNAc is potentially a good energy and nitrogen source, one might hypothesize that GlcNAc uptake is a widespread phenotype among bacteria [3]. However, the mechanism of GlcNAc uptake and subsequently its metabolism machinery in the cytoplasm has been studied in only a few bacteria such as *Escherichia coli* [4], [5], *Bacillus subtilis* [6], [7], *Staphylococcus aureus* [8], *Vibrio furnissii* [9], and *Caulobacter crescentus* [10]. Upon uptake, in the cytoplasm GlcNAc may take two metabolic routs i.e., (i) phopshorylation to GlcNAc-6-phosphate followed by deacetylation by nagA and subsequently production of either fructose-6-phosphate or UDP-GlcNAc; or (ii) it may directly enter in to cell wall peptidoglycan biosynthesis pathway [9], [11]. The product of these pathways UDP-GlcNAc, is a ubiquitous and essential metabolite and plays important roles in several metabolic processes [12]. In bacteria, it is known as a major cytoplasmic precursor of cell wall peptidoglycan and the disaccharide moiety of some lipids [13]. In eukaryotes, it serves as the substrate for chitin synthase, whose product chitin is a essential structural component for fungal cell wall [14]. It is also used in the GlcNAc moiety of N-linked glycosylation and the GPI-anchor of cellular membrane proteins [15].

The enzyme *N*-acetylglucosamine-6-phosphate deacetylase (nagA; EC 3.5.1.25) is a member of the amidohydrolase superfamily and catalyzes the deacetylation of GlcNAc-6-phosphate to yield glucosamine 6-phosphate, the first committed step in the biosynthetic pathway to amino-sugar-nucleotides and GlcNAc utilization as a carbon source by the bacterium [16], [17]. Additionally, the deacetylation of GlcNAc is also important in lipopolysaccharide synthesis and cell wall recycling [18], [19]. Importantly, unlike eukaryotes, bacteria lack the ability to convert GlcNac-6-phosphate to GlcNac-1-phosphate directly as they lack *N*-acetylglucosamine-phosphate mutase (AGM) and therefore conversion to GlcN-6-phosphate is a prerequisite for conversion to GlcN-1-phosphate by phosphoglucosamine mutase, which can then be acetylated and uridylated to UDP-GlcNAc[20]. As a result, nagA plays a critical role in the metabolic pathway and thus is essential for growth and survival of the bacterium on GlcNAc as an alternative carbon source. The nagA structure has been resolved in bacteria recently and it was found that the enzyme is a tetramer and requires Zn2^+^ in the native protein [21], [22]. Additionally, given its position at the crossroads of these key metabolic processes, nagA has warranted attention as a potential drug target. Indeed, sugar deacetylation is a validated therapeutic target in some other contexts [23], [24].

Like *E. coli* and other bacteria, the cellulose producing gram negative bacterium *Glucoacetobacer xylinus* can also metabolize GlcNAc and glucosamine as a carbon source although the preferred carbon source for *G. xylinus* is glucose. Metabolic pathways related to GlcNAc assimilation is well studied in *E. coli* and other bacteria [20], [25] but the precise GlcNAc metabolic cascade is not known in this commercially important bacterium. It was believed that in *G. xylinus,* GlcNAc-6-phosphate is first deacetylated to Gln-6-phosphate by nagA and subsequently converted to either fructose-6-phosphate or UDP-GlcNAc through a series of enzymatic steps. Prior to this report, there was no experimental evidence for the existence of this pathway in *G. xylinus*. Thus, we demonstrate here that like other bacteria, *G. xylinus* also possesses a similar mechanism for GlcNAc assimilation as an alternative sugar source. This is essential information with regards to our related work with the production of a GlcNAc-glucose heteropolymer from metabolically engineered *G. xylinus* [26]. In present work, we elucidate the GlcNAc metabolic machinery in *G. xylinus* strain10245 and demonstrated the role of nagA in GlcNAc metabolism by cloning a DNA fragment encoding a nagA and subsequently generating a nagA-deficient mutant by homologous recombination. The resulting knockout strain (nagA::tet^r^) was examined for growth, cytoplasmic UDP-GlcNAc pool, and overall cellulose productivity with glucose and/or GlcNAc as a carbon source. The successful deletion of this gene and the subsequent analysis provides a clearer picture of the related metabolic pathways of this potentially important biosynthetic pathway.

## Materials and Methods

### Bacterial strains, culture media and growth conditions

All bacterial strains, plasmids used in this study are listed in Table 1. The cellulose producing bacterium *G. xylinus* (ATCC strain 10245) and the nagA homolog-deficient mutant strain (ΔnagA; generated in this study) were used throughout this work. For cloning purposes, *E. coli* Top10 cells (Invitrogen) were used as a cloning host. Transformants of Top10 strains harboring various plasmids were cultivated at 37°C in LB medium containing either kanamycin (50 µg/ml) or ampicillin (50 µg/ml) or both. Unless otherwise mentioned, *G. xylinus *was cultured in Hestrin and Schramm (HS) medium (0.5% yeast extract, 0.5% peptone, 0.27% Na_2_HPO_4_, 0.15% citric acid, pH 4.5) supplemented with 2% sugar (glucose, unless otherwise noted) and grown at 30°C shaking at 200 rpm or statically [27]. The modified HS medium supplemented with micro-filtered celluclast cellulase (Sigma) to a concentration of 0.1% (v/v) was employed to culture *G. xylinus* without cellulose.

**Table 1:** Bacterial Strains and plasmids used in this study.

Strains or plasmid	Relevant genotype or description	Source and purpose
Strains		
*G xylinus* 10245	Wild type (wt)	Laboratory collection, This study
*G xylinus*ΔNAgA	ATCC10245 NagA::tet^r^	This study
E coli TOP10	F- mcrA Δ(mrr-hsdRMS-mcrBC) φ80lacZΔM15 ΔlacX74 nupG recA1 araD139 Δ(ara-leu)7697 galE15 galK16 rpsL(Str^R^) endA1 λ^−^	Invitrogen; Cloning
Plasmids		
pTOPO-Zero® blunt	Amp^r^ and Kan^r^; PCR cloning vector	Invitrogen; Cloning
pTOPO-NagA	Amp^r^ and Kan^r^; recombinant plasmid harboring nagA	This study
pT7-Blue®	Amp^r^; cloning vector	Novagen; Cloning
pT7-blue-NagA	Amp^r^; recombinant plasmid harboring NagA	This study
pT7-blue-NagA-tet-NagA	Amp^r^andTet^r^; recombinant plasmid harboring tetracycline flanked by nagA	This study

Amp^r^, ampicillin resistance; Kan^r^, Kanamycin resistance; Tet^r^, tetracycline resistance.

### Amplification, cloning and sequence analysis of the nagA

All primers used in this study are outlined in Table 2. Chromosomal DNA from strain 10245 grown on HS medium was extracted by using a PureLink™ genomic DNA extraction kit (Invitrogen). Degenerate PCR primers for amplification of a partial region of nagA were designed based on the nucleotide sequence comparison among bacteria belonging to the proteobacteria: *G. oxydans* 621H (nagA, GenBank YP_191872) and *G. diazotrophicus* PAl5 (nagA, GenBank NC_010125). After selecting the conserved regions utilizing the ClustalW algorithm (http://www.ebi.ac.uk/Tools/clustalw2/index.html), degenerate primers NagA-For and NagA-Rev were designed. PCR was performed according to standard procedures with 50 ng genomic DNA as template and 3Units of *pfu* taq polymerase (Invitrogen). The PCR program used was as follows: 95°C for 3 min (1 cycle); 95°C for 30 sec, 63°C for 30 sec, 72°C for 1 min (30 cycles) and 72°C for 5 min (1 cycle). The resulting amplified DNA fragments of approximately 0.5 kb were gel extracted (Qiagen). The purified DNA fragment was then ligated into pTOPO-Zero® blunt plasmid (Invitrogen) and was introduced into *E. coli* strain top10 by electroporation. The resulting plasmid pTOPO-NagA was extracted and sequenced. A homologous protein search was performed using pblastx algorithm (http://blast.ncbi.nlm.nih.gov/Blast). The deduced amino acid alignment of nagA from *G. xylinus* and other bacteria was performed using CLC-main workbench (version 5.5) algorithm (http://www.clcbio.com) The DNA sequence of the nagA homolog from strain 10245 was deposited into GenBank (accession number GU220906).

**Table 2:** Primers used in this study.

Name	5′-3′ Sequence	Purpose and restriction sites
NagA-For	CGCATGGCGTCSGTYACGAACAGCAG	Cloning
NagA-Rev	CATYCATGGCGCCATCTGGAAGG	Cloning
NagA-DisF	AGAAGCTTCATCCATGGCGCGCATCTGGAAG	Cloning; HindIII
NagA-DisR	TAGGATCCCGCATGGCGTCCGTTACGAACAG	Cloning; BamHI
Tetfor	TACATATGACTCATGTTTGACAGCTTATC	tet^r^ insertion in NagA; NdeI
Tetrev	ATCCATATGCCGGCTTCCATTCAGGTCGAG	tet^r^ insertion in NagA, NdeI
NagApcrF	TTGGCGCGCATCTGGAAGGGGCCGT	PCR confirmation
NagApcrR	TGGCGTCCGTTACGAACAGCAGCCGC	PCR confirmation

### Disruption of nagA

For nagA disruption, pT7-Blue plasmid (Navogen) was used as this plasmid had been reported to be used to successfully knock out a glucose dehydrogenase gene (gdh) from *G. xylinus* [28]. Initially, nagA was amplified by PCR with sequence specific primers (NagA-DisF and NagA-DisR) and inserted between the HindIII and BamHI sites in plasmid pT7-Blue. The resulting plasmid pT7-blue-NagA was further modified by the insertion of a tetracycline resistance cassette (tet^r^) into the NdeI site in between nagA sequence. For this, the tetracycline resistant gene was amplified using Tet-for and Tet-rev primers followed by digestion with NdeI. The NdeI digested tetracycline gene was finally ligated into NdeI digested pT7-blue-NagA plasmid. The resulting plasmid pT7blue-NagA-Tet-NagA was then introduced into *G. xylinus* by electroporation [29] and screened on HS agar plate containing 50 µg/ml tetracycline. In order to confirm the disruption of the nagA homolog, chromosomal DNA was extracted from potent recombinant colonies followed by PCR using primers (NagApcrF and NagApcrR). For the final confirmation, the resulting PCR amplified DNA fragment was sequenced and analyzed.

### Growth studies (ΔnagA v/s wild type)

Wild type and ΔnagA *G. xylinus* strains were cultured in 50 ml HS (+cellulase) medium supplemented with either glucose or GlcNAc. Initial optical density of the cell cultures was adjusted to A_600_ 0.01±0.005 and kept at 30°C with 200 rpm constant shaking. For growth, A_600_ was monitored at different time intervals up to 60 hrs. The obtained A_600_ values were plotted against respective culture time. For growth on solid media, HS-agar plates supplemented with either 2% glucose or 2% GlcNAc or 2% glucosamine were used. Both ΔnagA and wild type *G. xylinus* was streaked with sterile loop on the surface on agar plates. Plates were analyzed after 5 days and photographs were taken.

### Measurements of cytoplasmic UDP-GlcNAc and UDP-glucose

The cytoplasmic UDP-GlcNAc and UDP-glucose pools were measured by standard procedures [30]. Both ΔnagA and wild type cells were grown to mid logarithmic phase in the presence of either 2% glucose or 2% GlcNAc. Cultures were harvested at 3000 g for 10 min and the resulting cell pellets were mixed in 2 ml sonication buffer (100 mMKCl, 1 mM, EDTA, 50 mM KH_2_PO_4_, pH 7.5) followed by cell lysis using sonicator at 4°C. For sonication, 10 pulses (each pulse 60 sec) were applied to each sample with a 1 min gap between adjacent pulses. After sonication, samples were de-proteinized by addition of 1 volume of 0.1 M perchloric acid and subsequent centrifugation for 30 min at 16000 gat 4 °C for 30 min. The resulting supernatants were diluted with 10 volumes of 10 mM KH_2_PO_4_ (pH 2.5) and the final pH was adjusted to 2.5 for each sample. Samples were applied to 3-ml Supelclean LC-SAX solid-phase extraction columns (Supelco, USA). After the columns were washed with 5 ml of 10 mM KH_2_PO_4_ (pH 2.5) and 2.5 ml of 50 mM KH_2_PO_4_ (pH 2.5), the UDP-sugars were eluted with 1 ml of 150 mM KH_2_PO_4_ (pH 7.5). UDP-sugars were separated and quantified by HPLC (Waters), using two LC-18T columns in series (25 cm ×4.6 mm, 5 µm bead size; Supelco, USA) and ultraviolet detection at 254 nm (2487 Dual λ Absorbance Detector; Waters). The mobile phase, at a flow of 1 ml/min, was a 0.1 M KH_2_PO_4_ buffer containing 2 mM tetrabutylammonium phosphate, pH 6.2. For the quantification purpose, five calibrators (0, 10, 25, 50, 100 µM) of UDP-sugars (UDP-glucose and UDP-GlcNAc) were analyzed before and after each set of run. All unknowns were quantified using these calibration curves and individual samples were normalized with the A_600_ of the cultures at the time of harvesting.

### Cellulose production, purification and yield

For cellulose mass production, cell suspension of ΔNagA and wild type cells were cultured separately in 10-cm Petri dishes containing 20 ml HS medium supplemented with either 2% glucose or 2% GlcNAc or a mixture of 1.5% glucose 0.5% GlcNAc and kept at 30°C for one week. The cellulose pellicles were purified by treating twice with a solution containing 2% SDS and 0.1% NaOH at 70°C for 4 hrs to remove the entrapped *G. xylinus* cells followed by several washes with de-ionized water [31]. Purified cellulose mats were used to assess the cellulose production efficiency by drying at 70°C for 30 hrs and dry weights were normalized with culture volume.

### Atomic force microscopy (AFM)

For AFM, gelatin treated mica disks (∼1 cm diameter) were used to immobilize the bacterium on it [32]. Samples of both mutant and wild type *G. xylinus* were prepared by scraping-off a small quantity of the bacteria from a culture plate with a sterile loop and transferred into a micro-centrifuge tube containing 500 µl of PBS (pH 7.4). After mixing, a 5 µl cell aliquot was spotted onto a gelatin treated mica disk and spread to a diameter of 4-6 mm. The sample was allowed to stand for 30 min, rinsed with deionized water, and allowed to dry for imaging. Cell morphology was observed by AFM (Veeco, Nanoscope III) in air. A 100 µm long silicon cantilever with a spring constant of 0.6 N/m was used in tapping mode with scan speed of 0.8–1.2 Hz at 256 pixels per line [32].

### Acid hydrolysis of cellulose and quantitative analysis by LC-MS/MS

Both ΔnagA and wild type *G. xylinus* cells were grown in 10 cm Petri dishes containing 20 ml HS medium supplemented with either 2% glucose or 2% GlcNAc. After one week, cellulose pellicles produced from both wild type and mutant cells were de-cellularized by treating twice with 2% SDS at 70°C for 4 hrs followed by several washings with deionized water. The purified cellulose mates were acid hydrolyzed with 77% H_2_SO_4_ at 4°C for one hour with gentle agitation followed by neutralization with Ba(OH)_2_ and final pH was adjusted between 5–6 [33]. The acid hydrolysates were subjected to liquid chromatographic separation on a Waters Atlantis dC18 column (150×2.1 mm, 5 µm, Waters, Milford, MA) by Agilent HPLC 1200 system (Agilent, Santa Clara CA). Mass spectrometric detection was performed on an API 3200 triple quadrupole instrument (Applied Biosystems) using multiple reaction monitoring (MRM). A TurboSpray interface with negative ionization mode was used. The precursor-to-product ion transitions m/z 220 → 119 for GlcNAc and 179 → 89 for glucose. The main MS working parameters were listed in **Table S1**. A linear calibration standard curve for glucose and GlcNAc ranging up to 100 µg/ml was set up for the quantification purpose (**Figure S1**).

### Statistical analysis

Comparisons between two experimental groups were performed using one-way ANOVA (GraphPad, InStat Software, La Jolla, CA). Group means were deemed to be statistically significantly when p<0.001. Plasmid maps were drawn using pDRAW32 software (http://www.acaclone.com).

## Results

### Cloning and sequence analysis of nagA

**Figure 1:**
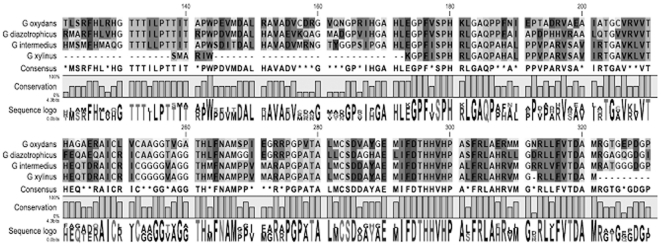
Multiple-amino-acid alignment of nagA proteins from *G. xylinus* and from other bacteria. Putative amino acid sequence of nagA homolog of *G. xylinus*, nagA of *G.intermedius* (Genbank access. no. BAI39462), nagA of *G. diazotrophicus* Pal 5 (YP_001602823), nagA of *G. oxydans* 621 H (YP_001602823).

A 460 bp DNA fragment was amplified by PCR from genomic DNA isolated from *G. xylinus* strain 10425 and sequenced. The deduced amino acid sequence showed significant similarities to nagA of several other proteobacteria (Fig. 1). The closest homology was the nagA of *G. intermedius* with a 97% amino acid sequence identity compare to other *Gluconacetobacer sp.* (Table 3). Given that our goal was to disrupt the gene to interrogate its effect on GlcNAc metabolism in *G. xylinus*, we did not require the full gene sequence. Therefore our research focus shifted to the disruption of the nagA gene and its consequential impacts in *G. xylinus*.

**Table 3:** GlcNAc-6-phosphate deacetylase (NagA) homology between *G. xylinus* and other bacteria.

Organism	Class	% Homology
*G. xylinum*	Alphaproteobacteria	100
*G. intermedius*	Alphaproteobacteria	97
*G. diazotrophicus*	Alphaproteobacteria	80
*D. geothermalis*	Deinococci	62
*D. radiodurans*	Deinococci	57
*S. thermophilum*	Clostridiales	60
*M. tuberculosis*	Actinobacteridae	59
*S. coelicolor*	Actinobacteridae	55

### Disruption of nagA

**Figure 2:**
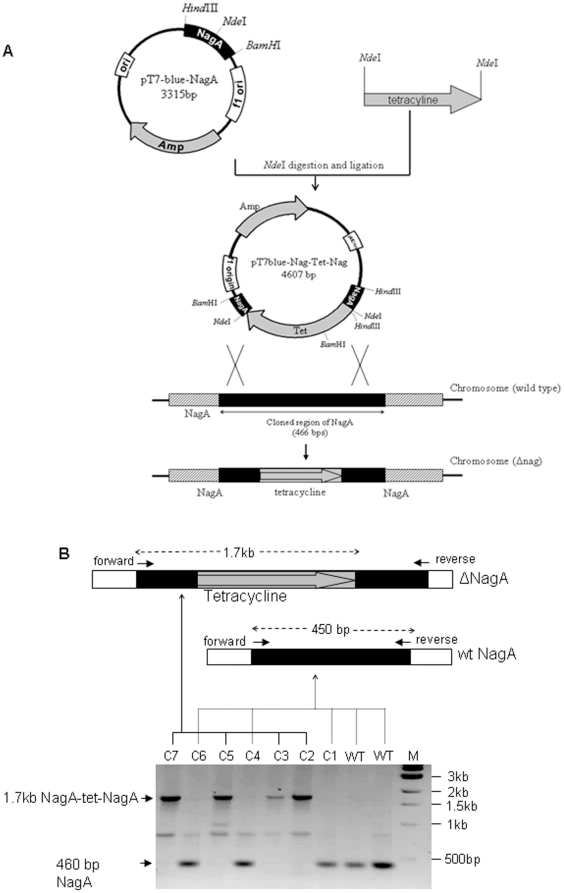
Disruption strategy and screening of *G. xylinus* nagA mutants. (A) pT7Blue-Nag-Tet-Nag was introduced in to *G. xylinus* strain 10245. Homologous recombination between the nagA homolog-flanking sequences in the plasmid and chromosomal DNA of strain 10245 occurred. (B) PCR screening of positive mutants using gene specific primers. These primers amplify 450 bp of wild-type genomic copies of nagA (wt, C1, C4 and C6) while in mutants nagA was efficiently disrupted (C2, C3, C5 and C7) gives a single 1.7Kb band as PCR product. Top pictures showing the possible genome organization of nagA in mutant (ΔnagA) and wild type cells (wt-nagA) to explain PCR data.

In order to generate a nagA-deficient strain we employed an insertional disruption technique. Initially the nagA sequence was cloned into the pT7-Blue plasmid between the BamHI and HindIII sites followed by insertion of a tetracycline resistance cassette in nagA sequence at the NdeI site. The resulting plasmid, pT7blue-NagA-Tet^r^-NagA is incapable of replicating in *G. xylinus*. Plasmids were introduced into strain 10245 by electroporation as previously described [29]. Since the plasmid lacks the *Ori* sequence (origin of replication) from *G. xylinus* and therefore cannot be maintained in *G. xylinus*, resistance to tetracycline will be present only after homologous recombination into the chromosome, presumably resulting in an insertional inactivation of the nagA gene (nagA :: tet^R^; ΔnagA). The complete procedure is outlined in Fig. 2A. After screening on tetracycline containing HS-agar plate, approximately two thirds of colonies were found to be sensitive to ampicillin (this means plasmid is no longer present in the cells) but resistant to tetracycline, thus suggesting that these recombinant strains were generated by a double crossover event. Disruption of the nagA homolog in the recombinant strains was confirmed by PCR with selected colonies. PCR analysis indicated that the ΔnagA strain generated a 1.7 kb amplicon (0.46 kb of nagA +1.25 kb of tet^r^) while the wild type strain generated a 0.46 kb amplicon in agarose gel (Fig. 2B). Sequence analysis of these PCR-amplified DNA fragments further confirmed the insertion of thetet^r^cassette into the NagA gene. While several disrupted clones were generated, a single strain was designated as a nagA deficient mutant (ΔnagA) and utilized for further studies.

### Measurements of cytoplasmic UDP-GlcNAc and UDP-glucose

**Figure 3:**
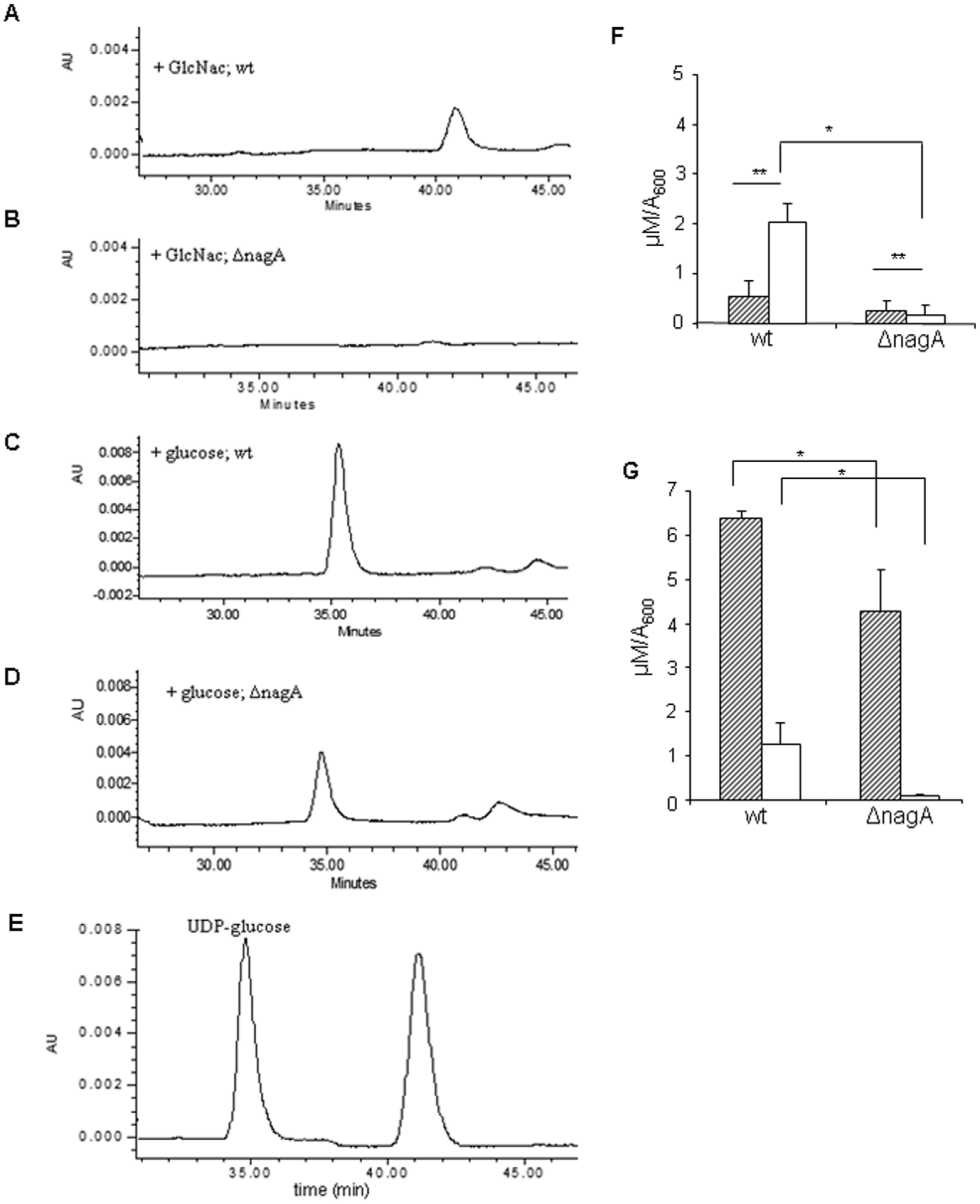
UDP sugar in wild type and ΔnagA cells. (A) HPLC elution profile for ΔnagA cells fed with either glucose or (B) GlcNAc. (C) HPLC elution profile for wild type cells fed with either glucose or (D) GlcNAc. (E) HPLC elution profile for purified UDP-glucose and UDP-GlcNAc as reference to evaluate the elution time for both. (F) Quantification of cytosolic level of UDP-glucose and UDP-GlcNAc in both nagA mutant and wild type *G. xylinus* cells fed either GlcNAc or (G) glucose fed conditions. Error bar represent s.d. of three replicates (p<0.001).

To determine whether ΔnagA cells are able to metabolize GlcNAc, cytoplasmic UDP-GlcNAc and UDP-glucose were assessed by HPLC in ΔnagA (Fig. 3A, 3B) and (Fig. 3C, 3D) and normalized with relative cell numbers by measuring A_600_ of cultures at the time of harvesting (Fig. 3E for ΔnagA and Fig. 3F for wild type sells respectively). Purified UDP-glucose and UDP-GlcNAc were used as standard (Fig. 3E). In the presence of GlcNAc, wild type cells contains significant level of UDP-GlcNAc (2.12±0.45 µM/A_600_) compare to mutant cells having almost undetectable level of UDP-GlcNAc (Fig. 3F). Under glucose fed conditions, levels of UDP-glucose observed in wild type cells (6.3±0.4 µM/A_600_) were slightly higher than ΔnagA cells (4.24±1.16 µM/A_600_) (Fig. 3G).

### Growth studies and phenotypic appearance

**Figure 4:**
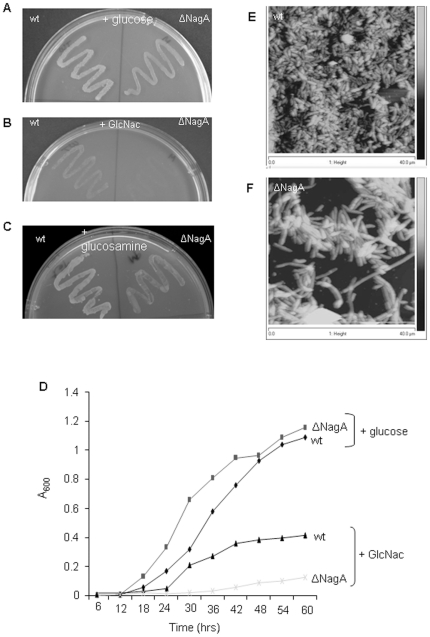
Growth and morphology of wild type and ΔNagA mutants. (A) growth on HS-agar plate supplemented with either glucose; or (B) glucosamine or (C) GlcNAc. (D) Time dependent growth curve of wild type and mutant cells in the presence of glucose or GlcNAc as carbon source. (E) AFM imaging of wild type and (F) nagA mutant cells.

To determine whether nagA is essential for growth of the bacterium, both ΔnagA and wild type *G. xylinus* were grown for five days on HS agar plate supplemented with either glucose, GlcNAc, or glucosamine. On glucose and glucosamine supplemented plates, both mutant and wild type grew well (Fig. 4A and Fig. 4B) while on GlcNAc supplemented plates, mutant cells did not grow (Fig. 4C). To confirm these data, the growth of both wild type and mutant cells was monitored in liquid HS media supplemented with glucose or GlcNAc. In glucose-supplemented media, both mutant and wild type cells exhibited a typical sigmoidal growth pattern whereas in the presence of GlcNAc, growth of mutant cells was completely inhibited while wild type cells grew slowly (short log phase) as expected due to the fact that GlcNAc is not a preferred carbon source for *G. xylinus *(Fig. 4D). Additionally, in presence of glucosamine, growth of both wild type and ΔnagA mutant was similar to the growth of wild type in GlcNAc fed conditions (data not shown). These findings together with agar plate growth studies demonstrated that nagA disruption prevents *G. xylinus* from metabolizing GlcNAc as an alternative carbon source for growth. Similar findings were also observed in *Gluconacetobacter intermedius* where disruption of nagA decreased the growth rate in the exponential growth phase [34]. Nevertheless, a steady sate growth curve of mutant cells with GlcNAc feed also revealed that nagA disruption does not cause any lethal impact (lysis or death) on the bacterial cells and as a result it only impairs the growth. To conform the mutant cells (grown in presence of GlcNAc) were sub-cultured in the presence of glucose and found that bacterium regains its normal growth (data not shown). This reveals that mutant cells do not die in the presence of GlcNAc and only their growth has been inhibited. Atomic force microscopy was employed to analyze cell morphology and no significant changes were observed between wild type (Fig. 4E) and mutant cells (Fig. 4F).

### Cellulose Productivity and chemical composition

**Figure 5:**
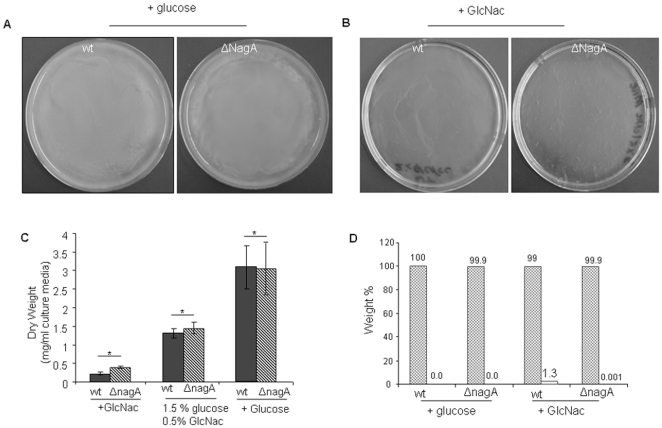
Cellulose production and pellicle morphology. (a) Morphological appearance of cellulose pellicles produce after one week by wild type and ΔNagA *G. xylinus* in the presence of either (A) glucose; or (B) GlcNAc as carbon source. (C) Cellulose production efficiency of wild type and ΔNagA *G. xylinus* supplemented with either glucose, GlcNAc or both as carbon source. Error bar represent s.d. of three replicates (*p<0.001); (D) Quantitative LC-MS/MS for quantification and relative weight percent (wt%) of glucose and GlcNAcin acid hydrolyzed cellulose extruded from wild type or ΔNagA fed with either glucose or GlcNAc. Error bar represent s.d. of three replicates (*p<0.001). (E) Linear regression curve for known glucose; and (F) for known GlcNAc to quantify glucose and GlcNAc in acid hydrolyzed cellulose samples by LC-MS/MS.

The gross morphology of cellulose pellicles produced by wild type or ΔnagA cells was not noticeably different (Fig. 5A and Fig. 5B). To completely assess cellulose production efficiency in the mutant strain, the cellulose yield was determined by measuring dry weight of pellicles and compared with cellulose dry weight from wild type cells. The cellulose yield in presence of glucose was found to be 3.1±0.4 mg/ml-culture media for wild type and 3.0±0.6 mg/ml-culture media for ΔNagA. In the presence of GlcNAc media supplement, cellulose yields were 0.31±0.1 mg/ml-culture media for wild type and 0.52±0.1 mg/ml-culture media for ΔNagA. Moreover, as we increased glucose content in culture medium, the cellulose yield also increased accordingly for both wild type and mutants. To conclude these findings, we did not observe any loss of cellulose productivity in mutant cells when compared to the wild type strain (Fig. 5C). This is extremely important for further exploration of the nagA mutant strain in studies where cellulose productivity cannot be compromised. The cellulose composition was determined by acid hydrolysis followed by LC-MS/MS (Fig. 5D). Under glucose fed conditions, cellulose produced from either wild type or ΔnagA was essentially pure and was made-up of glucose moieties only while in presence of GlcNAc, mutant cells produces pure cellulose (99.9 weight% glucose) but cellulose from wild type cells contains 1.3 weight% GlcNAc as previously reported [35]. Additionally, our MS data showed that there were no significant changes in enzymatic digestion pattern as mass spectra for cellulase-digested cellulose from ΔnagA is identical to wild type cellulose. This indicates that deletion of NagA alone does not alter the molecular composition of cellulose.

## Discussion

**Figure 6:**
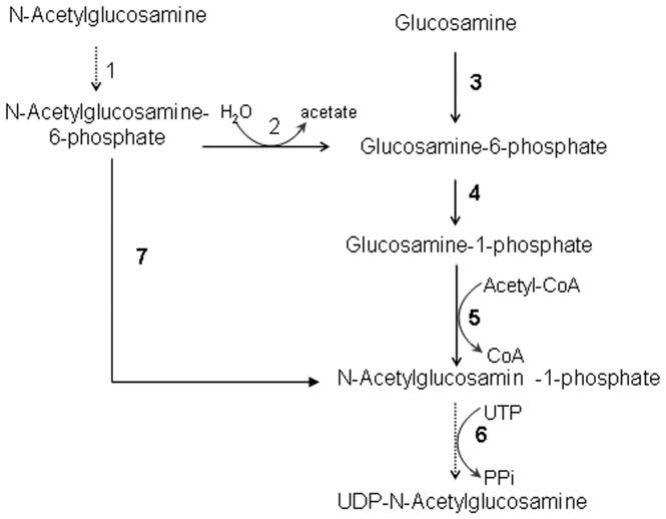
UDP-GlcNAc biosynthetic pathway in bacteria (including *G. xylinus*) and eukaryotes. Reactions with open arrows occur in bacteria and reactions with filled arrow occur in eukaryotes while dotted arrow reactions are common in both bacteria and animals. Enzymes involved are as follows; 1: N-acetylglucosamine kinase; 2: N-acetylglucosamine-6-phosphate deacetylase; 3: Glucosamine kinase; 4: Phosphoglucosamine mutase; 5: Glucosamine-6-phosphate N-acetyltransferees; 6: UDP-N-acetylglucosamine pyrophosphorylase; 7: Phosphoacetylglucosamine mutase.

In the present study, we report a genomic DNA fragment belonging to N-acetylglucosamine 6-phosphate deacetylase (nagA) from cellulose producing bacterium *G. xylinus*. In *E. coli* and other prokaryotes, nagA is demonstrated to be involved in GlcNAc metabolisms by deacetylating GlcNAc-6-P to Gln-6-P [6], [22], [36], [37]. Therefore we sought to investigate the role of nagA in N-acetylglucosamine assimilation in *G. xylinus* by disrupting nagA. Since nagA mutants were able to grow on glucosamine, this clearly indicates that deacetylase is not involved in glucosamine degradation. Due to the fact that UDP-GlcNAc was almost undetectable in ΔnagA cells under either glucose or GlcNAc feed, we not only conclude that nagA is essential for conversion of GlcNAc supplements in to UDP-GlcNAc but also that *G. xylinus* lacks the enzyme AGM, as in presence AGM bacteria would be able to synthesize UDP-GlcNAc even in the absence of nagA. Based on our results and the GlcNAc metabolic pathway from both prokaryotes and eukaryotes, we believed that following steps occurred in *G. xylinus*: i) conversion of GlcNAc-6-phosphate into glucosamine-6-phosphate (GlcN-6-P) by NagA; ii) conversion of GlcN-6-P into glucosamine-1-phosphate (GlcN-1-P); iii) acetylation of GlcN-1-P to produce *N-*acetylglucosamine-1-phosphate (GlcNAc-1-P); and iv) synthesis of UDP-GlcNAc from GlcNAc-1-P and UTP. The overall pathway is illustrated in Fig. 6. The growth characteristics of deacetylaseless mutants confirm the catabolic routes for glucosamine and GlcNAc in *G. xylinus*. The inhibited growth of mutants in presence of GlcNAc was due to lack of adequate UDP-GlcNAc in cytoplasm for peptidoglycan cell wall synthesis; as a result the bacteria could not multiply.

Earlier studies have shown that *G. xylinus* is able to incorporate GlcNAc in cellulose while grown under GlcNAc fed conditions [31],[35]. Therefore we sought to evaluate the chemical composition of cellulose produced by both wild type and mutant cells and the role of nagA in this procedure. We did not observe any GlcNAc content in cellulose produced by mutant cells while a small fraction of GlcNAc was observed in wild type cells as reported earlier [35]. The absence of UDP-GlcNAc in nagA mutant cells translates into the absence of GlcNAc residues in cellulose produced from ΔnagA cells even under GlcNAc fed conditions.

Bacterial cellulose (BC) produced by *G. xylinus* into long, non-aggregated, essentially pure nanofibrils and is a versatile biomaterial due to its unique nanostructure and properties that closely resemble the structure of native extracellular matrices [5], [38], [39]. Despite the excellent biocompatibility and mechanical properties of BC, the lack of cellulose hydrolyzing enzymes in the human body and the high crystallinity restricts its utility [40]. Therefore, cellulose with controllable crystallinity and degradability (in the human body) could be a next generation polymer for tissue engineering applications. Nonetheless the widespread presence of lysozyme in human body warrants its exploitation to degrade a biopolymer containing GlcNAc as one of its constituent [41], [42]. Since the cellulose synthase of *G. xylinus* can utilize both UDP-glucose and UDP-GlcNAc as substrate further genetic alteration can be carried out in *G. xylinus* to elevate the UDP-GlcNAc pool. This would make UDP-GlcNAc accessible for cellulose synthase and as a result such cells may produce a lysozyme degradable cellulosic heteropolymer consisting of both glucose and GlcNAc. In this regard, our group has published a report on the production of cellulose chitin copolymer from metabolically engineered *G. xylinus* [26].Though we were able to incorporate GlcNAc (21% GlcNAc/dry weight) in cellulose produced from metabolically engineered cells but still we believe that elevated cytoplasmic UDP-GlcNAc (as its high level could harm *G. xylinus* cells) could allosterically activate nagA to circumvent the GlcNAc-6-phosphate into other metabolic pathways thereby reducing the level of cytoplasmic UDP-GlcNAc pool. Although disruption of NagA neither induces incorporationof GlcNAc into cellulose nor changes cellulose composition, simultaneous disruption of NagA and heterologous expression of UDP-GlcNac synthesis machinery would likely increase the cytoplasmic UDP-GlcNAc pool. Based on that context, the amount of GlcNac incorporated into cellulose would likely be higher than what was previously achieved [26]. We anticipate that this would lead to the production of a cellulosic heteropolymer consisting of both glucose and GlcNAc as its constituents thereby producing a tailorable (due to presence of polar GlcNAc) chimeric cellulosic biopolymer degradable in human body. This could overcome the longstanding limitations associated with the *in vivo* cellulose degradability.

## Supporting Information

Figure S1 (A) Linear regression curve for known glucose; and (B) for known GlcNAc to quantify glucose and GlcNAc in acid hydrolyzed cellulose samples by LC-MS/MS.
(TIFF)

Table S1 Mass spectrometer main working parameters for glucose and GlcNAc quantitative analysis in acid hydolysates.
(DOCX)
