# Supplementary figures and images for: N-acetylglucosamine 6-Phosphate Deacetylase (nagA) Is Required for N-acetyl Glucosamine Assimilation in Gluconacetobacter xylinus
Source: PLoS One. 2011 Jun 2;6(6):e18099. doi: 10.1371/journal.pone.0018099 (PMC3107205; doi:10.1371/journal.pone.0018099)

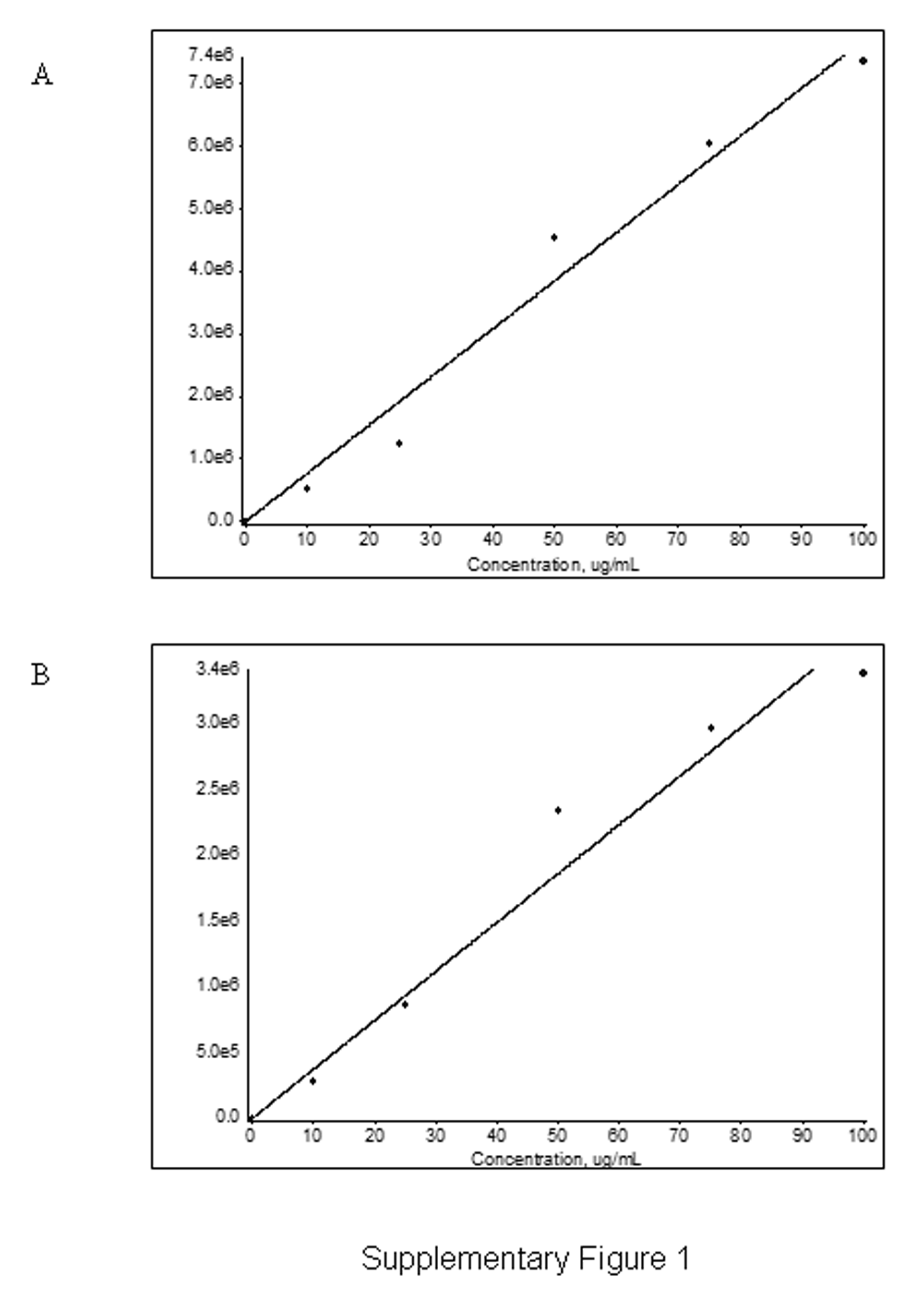

Supplement: Figure S1 — (A) Linear regression curve for known glucose; and (B) for known GlcNAc to quantify glucose and GlcNAc in acid hydrolyzed cellulose samples by LC-MS/MS. (TIFF) [file pone.0018099.s001.tiff]
