# Supplementary material for: N-acetylglucosamine 6-Phosphate Deacetylase (nagA) Is Required for N-acetyl Glucosamine Assimilation in Gluconacetobacter xylinus
Source: PLoS One. 2011 Jun 2;6(6):e18099. doi: 10.1371/journal.pone.0018099 (PMC3107205; doi:10.1371/journal.pone.0018099)
Supplement: Table S1 — Mass spectrometer main working parameters for glucose and GlcNAc quantitative analysis in acid hydolysates. (DOCX) [file pone.0018099.s002.docx]

**Table S1:** Mass spectrometer main working parameters for glucose and GlcNAc quantitative analysis in acid hydolysates

| **Parameter** | **value** |
| --- | --- |
| Declustering Potential (DP, V) | -30 |
| Entrance potential (EP, V) | -10 |
| Collison Energy (CE, V) | -11 |
| Collison Cell exit Potential (CXP,V) | -2 |
| Curtain Gas (CUR) | 11 |
| Collision Gas (CAD) | medium |
| Ion Spray Voltage (IS, V) | -2600 |
| Temperature (℃) | 350 |
| Ion Sourse Gas 1 (GS1) | 20 |
| Polarity of anaslysis | negative |
| Ion transition for GlnNac, m/z | 220/119 |
| Ion transition for Glucose, m/z | 179/89 |
